# Risk factors for residual lesions after total hysterectomy in patients with high-grade cervical intraepithelial neoplasia

**DOI:** 10.1186/s12905-024-03212-x

**Published:** 2024-06-24

**Authors:** Jing Wang, Chao Wang, Tao Su

**Affiliations:** 1 The International Peace Maternity and Child Health Hospital, School of Medicine, Shanghai Jiao Tong University, Shanghai, 200030 China; 2 Shanghai Key Laboratory of Embryo Original Diseases, Shanghai, 200030 China

**Keywords:** Cervical intraepithelial neoplasia, Positive margin, Glandular involvement, ECC, Vaginal intraepithelial neoplasia, Hysterectomy

## Abstract

**Background:**

The purpose of this study was to predict the risk factors for residual lesions in patients with high-grade cervical intraepithelial neoplasia who underwent total hysterectomy.

**Methods:**

This retrospective study included 212 patients with histologically confirmed high-grade cervical intraepithelial neoplasia (CIN2-3) who underwent hysterectomy within 6 months after loop electrosurgical excision procedure (LEEP). Clinical data (e.g., age, menopausal status, HPV type, and Liquid-based cytology test(LCT) type), as well as pathological data affiliated with endocervical curettage (ECC), colposcopy, LEEP and hysterectomy, were retrieved from medical records. A logistic regression model was applied to estimate the relationship between the variables and risk of residual lesions after hysterectomy.

**Results:**

Overall, 75 (35.4%) patients had residual lesions after hysterectomy. Univariate analyses revealed that positive margin (*p* = 0.003), glandular involvement (*p* = 0.017), positive ECC (*p* < 0.01), HPV16/18 infection (*p* = 0.032) and vaginal intraepithelial neoplasia (VaIN) I-III (*p* = 0.014) were factors related to the presence of residual lesions after hysterectomy. Conversely, postmenopausal status, age ≥ 50 years, ≤ 30 days from LEEP to hysterectomy, and LCT type were not risk factors for residual lesions. A positive margin (*p* = 0.025) and positive ECC (HSIL) (*p* < 0.001) were identified as independent risk factors for residual lesions in multivariate analysis.

**Conclusions:**

Our study revealed that positive incisal margins and ECC (≥ CIN2) were risk factors for residual lesions, while glandular involvement and VaIN were protective factors. In later clinical work, colposcopic pathology revealed that glandular involvement was associated with a reduced risk of residual uterine lesions. 60% of the patients with residual uterine lesions were menopausal patients, and all patients with carcinoma in situ in this study were menopausal patients. Therefore, total hysterectomy may be a better choice for treating CIN in menopausal patients with positive margins and positive ECC.

## Highlights

Positive margins and positive ECC were found to be independent risk factors for residual lesions after hysterectomy for high-grade cervical intraepithelial neoplasia.

Glandular involvement should be indicated in colposcopic pathology.

Any type of positive margin should be further treated.

## Introduction

Cervical cancer is the final stage of gradual progression from cervical intraepithelial neoplasia (CIN), and is closely related to persistent high-risk human papillomavirus (HR-HPV) infection; however, this process of progression is not present in all cases. According to the Global Cancer Report 2022, cervical cancer ranks fourth in both incidence and mortality among female malignancies, with 659,600 new cases and 348,300 deaths [1]. The detection and treatment of CIN before its progression to cancer are key to reducing the incidence of cervical cancer [2, 3].

Cervical intraepithelial neoplasia (CIN) can be divided into three grades based on severity: CIN1-3 indicated severe lesions, CIN3 also contains carcinoma in situ [4]. Patients with high-grade CIN (CIN2-3) are more willing to undergo the loop electrosurgical excision procedure (LEEP) as a treatment and diagnostic method because of its rapid effect, minimally invasiveness and lower incidence of postoperative complications [5–8], but previous studies have shown that patients who undergo the LEEP are more prone to cervical tube contracture, occlusion, cervical insufficiency and residual lesions at the resection margin [9–11]. Regardless of the CIN grade, residual lesions after LEEP are predictors of disease persistence [12]. The percentage of positive LEEP residue was as high as 12–26% [9, 10, 13, 14]. Some studies have confirmed that the risk factors associated with CIN 2/3 residue include residual margin, lesion size, lesion severity and location, depth and method of excision, patient age and menopausal status, and the presence of HR-HPV after excision [14–20].

For patients with positive margins, follow-up, repeat diagnostic excisional procedures or direct total hysterectomy can be selected according to age and actual conditions. However, residual intraepithelial neoplasia caused by excision of CIN 3 was found in 29–57% of patients who subsequently underwent hysterectomy. Hence the primary objective of this study was to assess the risk factors for residue positivity in patients with high-grade cervical intraepithelial neoplasia who underwent total hysterectomy.

## Materials and methods

**Fig. 1:**
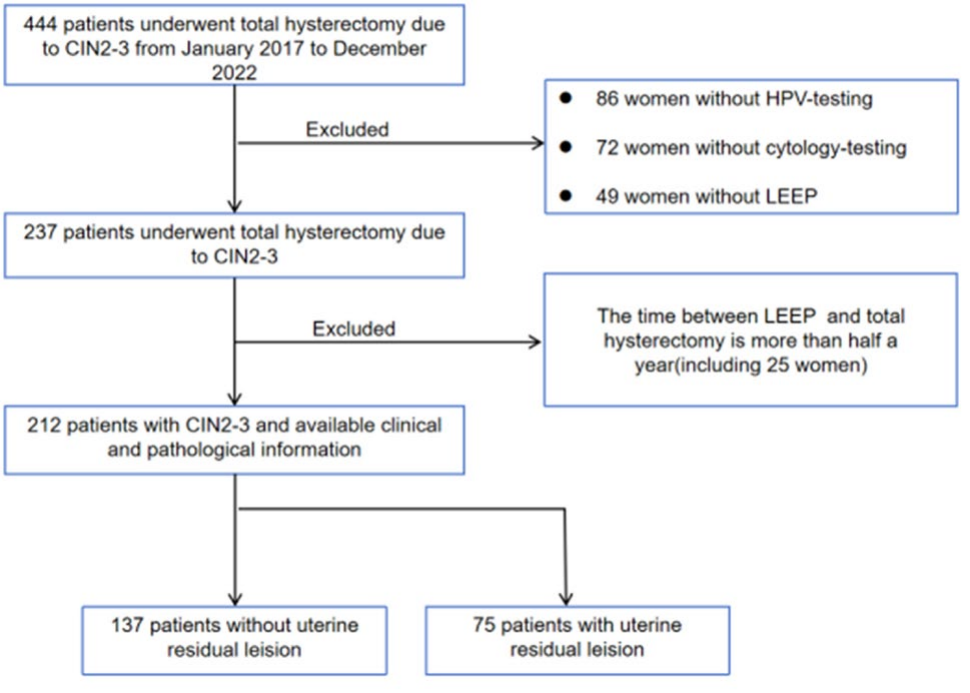
Study flow chart

This retrospective study included patients who underwent total hysterectomy for high-grade CIN between January 2017 and December 2022 in the International Peace Maternity and Child Health Hospital (IPMCH), Shanghai Jiao Tong University School of Medicine. Clinical data such as age, cytology results, HPV genotype testing, endocervical curettage (ECC), colposcopy, LEEP, and hysterectomy pathology were obtained from patients’ medical records. Women who desired to have children, wished to preserve their uterus, or whose clinical documentation was incomplete were excluded from this study. A flow chart is shown in Fig. 1.

### LCT and HR-HPV screening

Liquid-based cytology test(LCT) was performed for cytological examination of the cervical region, and the Bethesda System standard 2014 [4] was used for the cytological classification: (1) normal; (2) atypical squamous cells of unknown significance (ASC-US); (3) atypical glandular cells (AGCs); (4) atypical squamous cells, cannot exclude an HSIL (ASC-H); (5) low-grade squamous intraepithelial lesion (LSIL); (6) high-grade squamous intraepithelial lesion (HSIL); (7) squamous cell carcinoma(SCC); and (8) adenocarcinoma (AC).

The HR-HPV results showed the qualitative results for HPV-16, 18 and the other 12 high-risk HPV subtypes.

The LCT and/or HR-HPV test results were referred to as the results at the last follow-up or when CIN was diagnosed.

### Colposcopy and LEEP for pathological examination

Colposcopy(TRME CH7000, China) was performed when the results of the corroborative test were abnormal. Patients with no abnormalities in leucorrhea could undergo colposcopy, and after staining with acetic acid and iodine reagents, the suspected lesions were biopsied according to colposcopic images. Then, LEEP(TRME Power 420A6, China) was performed according to the pathology of the colposcopy biopsy within 3 to 7 days after menstruation. Excision was performed according to the patient’s transformation area and lesion location at colposcopy.

### Hysterectomy

All the hysterectomies were performed either laparoscopically or vaginally. The pathological results of the patients were recorded.

### Important definitions

In our study, the incisal margin of LEEP specimens with cervical intraepithelial neoplasia (≥ CIN2) or invasive cancer was defined as margin-positive. We performed ECC during colposcopy biopsy, not during LEEP. At the same time, positive ECC was defined as ≥ CIN2-positve. Residual uterine lesions were characterized by CIN ≥ 2 in hysterectomy specimens within 6 months after LEEP.

### Statistical analysis

SPSS version 22.0 (IBM Corp., Armonk, NY, USA) was used for the data analysis. Quantitative data are summarized as means and standard deviations. Categorical variables are expressed as counts or percentages. For comparison, we used age ≥ 50 years and time from the LEEP to hysterectomy ≤ 30 days as cutoff values. Chi-square tests were used compare categorical data, such as the LEEP excision margin report (the negative excision margin group included chronic cervical inflammation. Positive margins include the endo-cervical margin, ecto-cervical margin, stromal margin and two or more margins). Logistic regression analysis was used to evaluate the predictors of high-grade CIN residual lesions after hysterectomy and to construct a clinical prediction model. Multivariate logistic regressions were used to calculate the odds ratios (ORs) and 95% CIs after simultaneously controlling for potential confounders. All p values were two-tailed, with a *p value* < 0.05 considered to indicate statistical significance.

## Results

### Clinical features of the patients

A Total of 212 patients were enrolled in the study. All patients underwent total hysterectomy for high-grade CIN and had detailed information about HPV, LCT, colposcopy and LEEP (Table 1). The 212 patients, with a mean age of 55 years, had a median time of 41 days from LEEP to total hysterectomy. As shown in Tables 1 and 36.6% of the patients were menopausal. A total of 62.3% of patients were HPV 16- or 18- positive, and 109 cases (51.4%) had LCT manifestations ≥ ASC-H. Among the patients with cervical LCT or HPV abnormalities who underwent colposcopy and ECC, 56.6% had positive ECC results, including HSIL. Among the patients who underwent colposcopy, 15 patients also had vaginal intraepithelial lesions (7 patients had VaIN2-3, 7 had VaIN1, and 1 had carcinoma in situ). The detailed information of these patients with vaginal lesions is shown in Table 2. Among the patients with abnormal colposcopic pathology who wnderwent LEEP, 145 (68.4%) had positive margins (74 patients had positive endo-cervical margins, 28 patients had ecto-cevical margins, 48 patients had stromal margins and 27 patients had more than two margins). Pathological results of the LEEP showed that 177 (83.5%) patients had glandular involvement. Moreover, the data of patients with cervical residual CIN1, CIN2, CIN3 and CIS are shown in Table 3. All CIS patients were postmenopausal, and 4 of them had positive incisal margins.

**Table 1:**
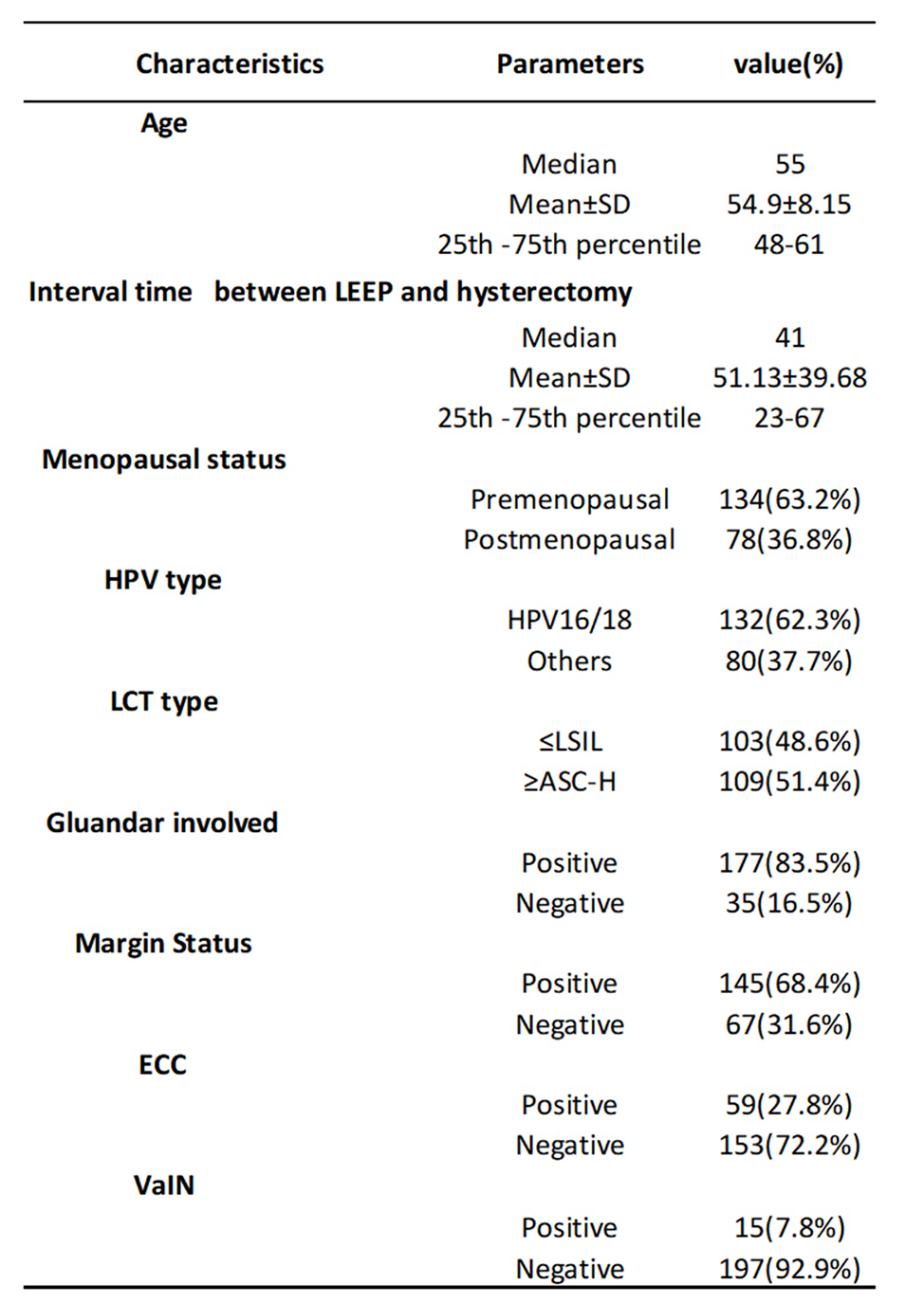
Baseline characteristics of all 212 patients

**Table 2:**
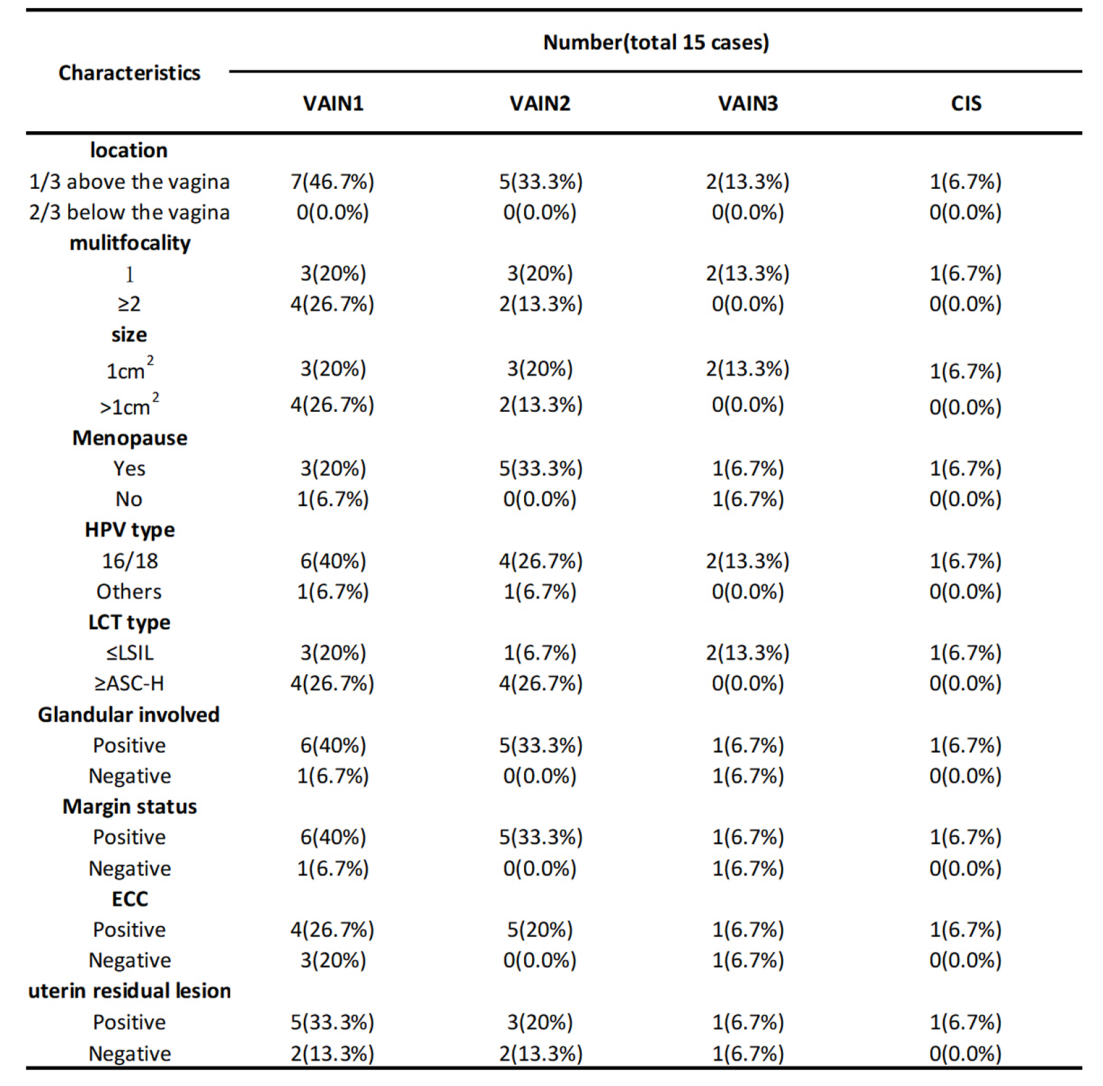
Characteristics of VaIN

**Table 3:**
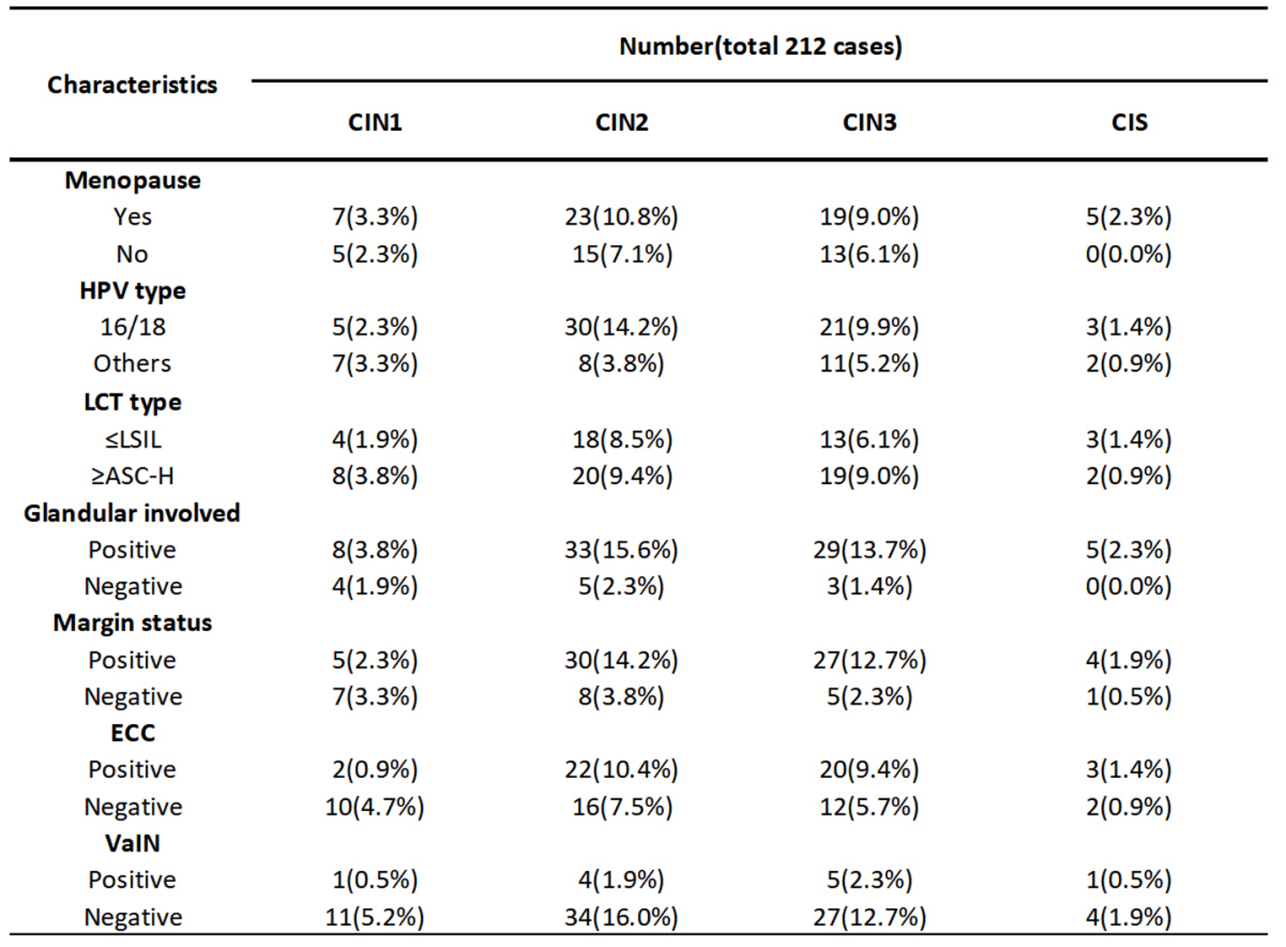
Characteristics of cervical CIN1, CIN2, CIN3 and CIS in hysterectomy specimens

### Univariate and multivariate analyses for predicting residual lesions after total hysterectomy

Univariate logistic regression analysis revealed that positive HPV16/18(*p* = 0.032), positive margin (*p* = 0.003), positive ECC (HSIL) (*p* < 0.01), VaIN (*p* = 0.032), and glandular involvement (*p* = 0.032) were associated with residual lesions (Table 4). In contrast, other parameters, such as menopause status, age, time from the LEEP to hysterectomy and LCT type, had no value for predicting residual lesions. Subsequently, we used multivariate logistic regression to analyse the presence of HPV16/18, positive margins, positive ECC, VaIN, and glandular involvement. Multivariate analysis revealed that a positive margin (*p* = 0.025), positive ECC (HSIL) (*p* < 0.001), VaIN (*p* = 0.003) and glandular involvement (*p* = 0.043) were independent factors related to the presence of residual lesions after hysterectomy (*p* < 0.05; Table 5).

**Table 4:**
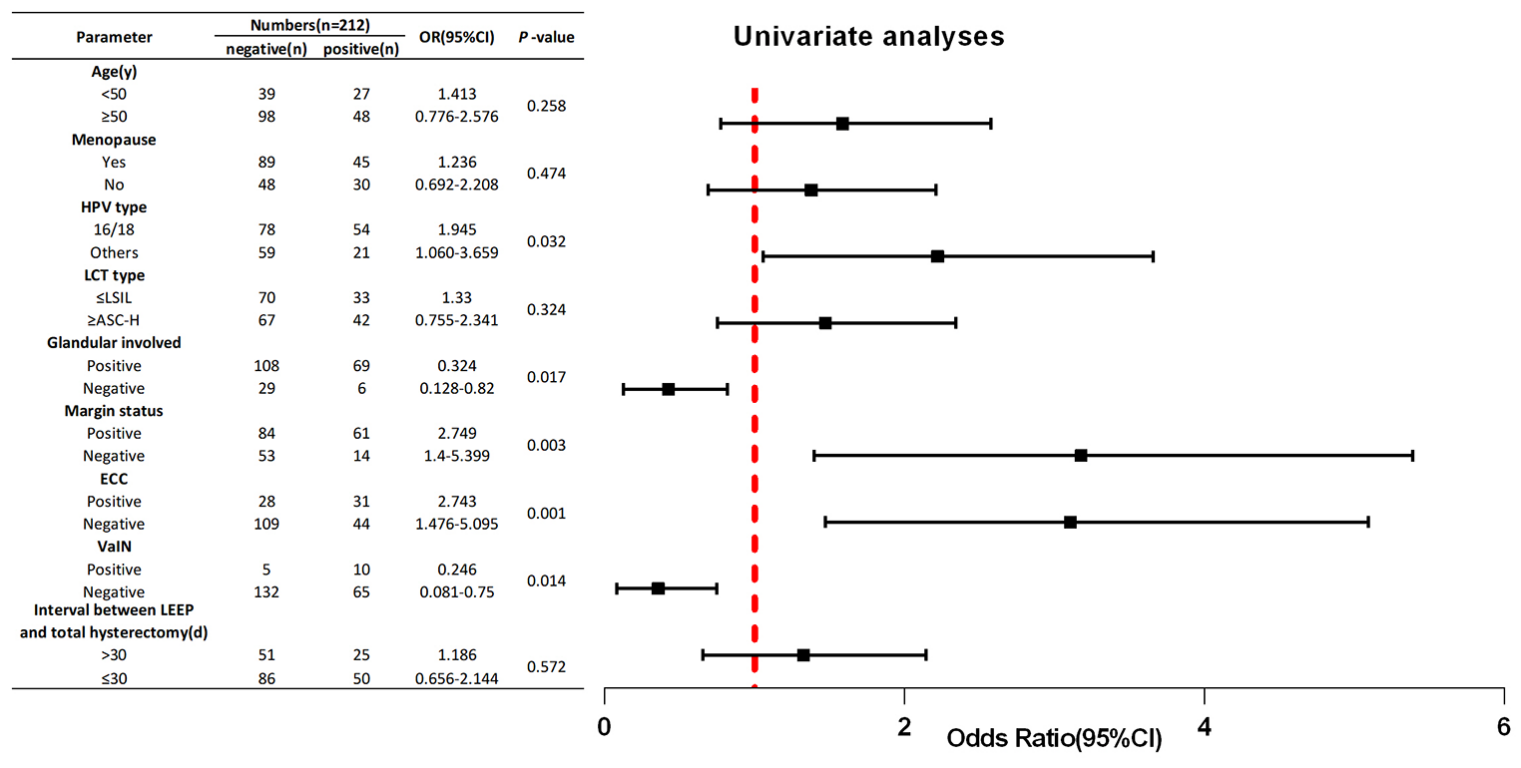
Univariate analyses for parameters related to residual disease in post-conization hysterectomy samples

**Table 5:**
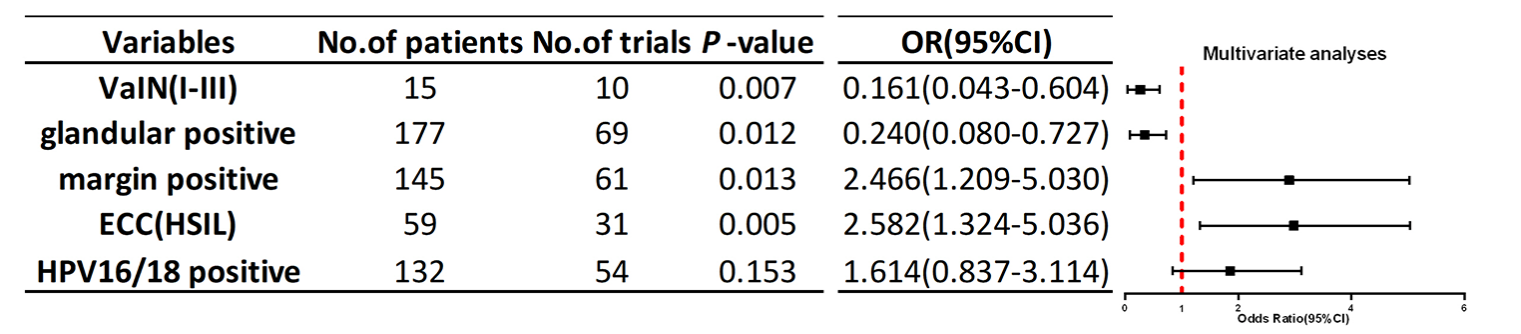
Multivariate analyses for variables related to residual disease in post-conization hysterectomy samples

### Analysis of risk factors for residual lesions after total hysterectomy with different positive margins

In our study, different positive incisal margins were recorded in detail, including endo-cervical, ecto-cervical, stromal and 2 or more positive margins. We compared the clinical data of patients with and without residual lesions in the uterus and found that the presence of an endo-cervical margin, an ecto-cervical margin, a stromal margin and more than two kinds of margins were risk factors for residual lesions in the uterus, but there was no significant difference in the influence of different incisal margins on residual lesions. (Table 6)

**Table 6:**
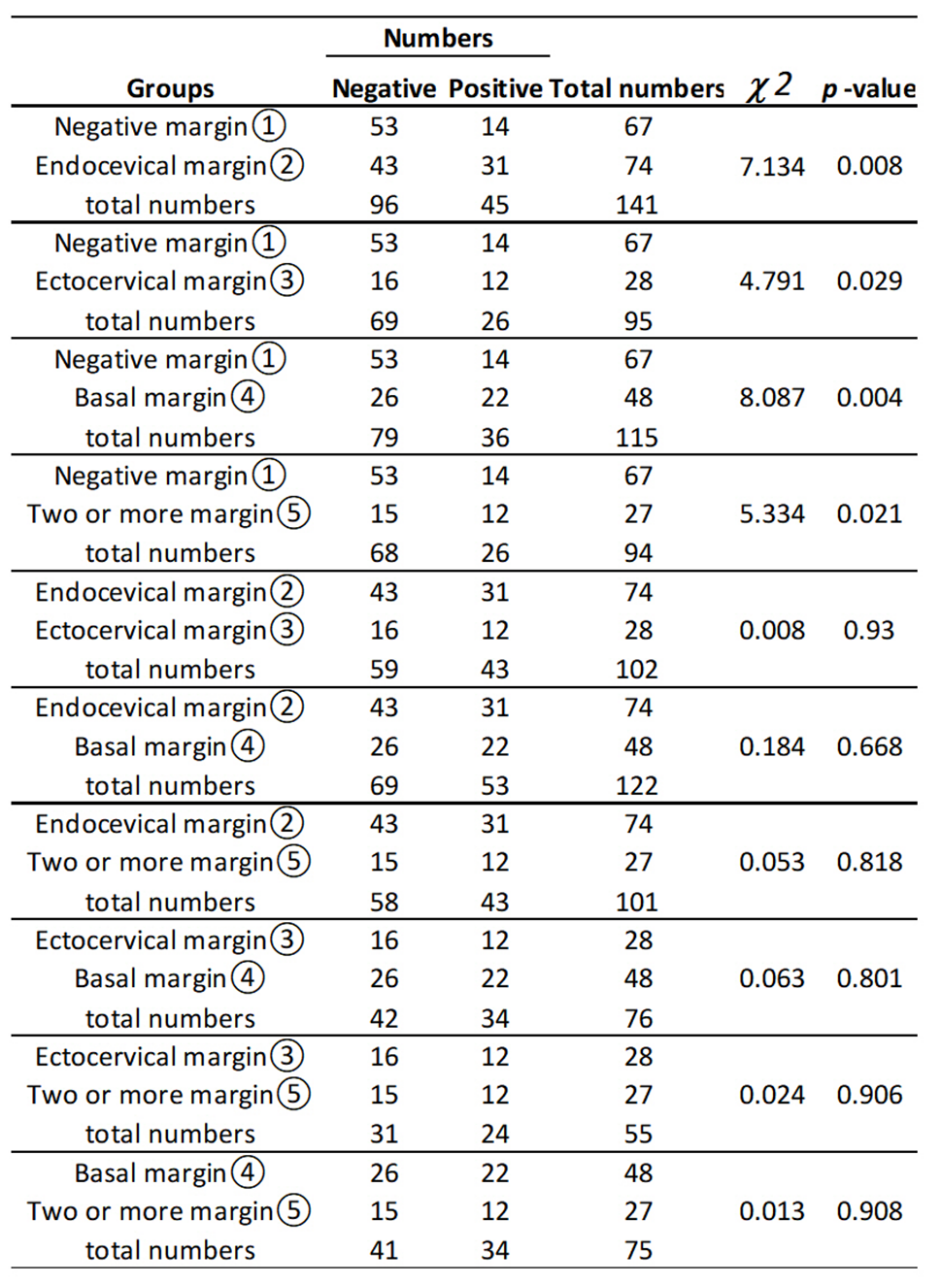
Analysis of risk factors for residual lesions after total hysterectomy with different positive margins

## Discussion

The purpose of this study was to explore the factors related to the presence of residual cervical lesions in patients who underwent total hysterectomy due to high-grade CIN. Moreover, it was confirmed that cervical ECC indicating ≥ CIN2, VaIN, glandular involvement and positive margins were independent factors related to the presence of residual cervical lesions.

Previous studies have confirmed that a positive LEEP margin is closely related to residual lesions of the uterus, in which 30–90% of residual lesions of the uterus have positive LEEP margins, and 83% have positive endo-cervical margins [10, 14, 20, 21]. In our study, the endo-cervical margin was positive in 51% (74/145) of patients, and we found that up to 68.4% (145/212) of patients had positive margins, while 81.3%(61/75) of patients with residual disease after hysterectomy had positive margins. This may be related to the fact that the average age of our patients was higher, and most of them were menopausal. Our study suggested that age and menopause were not associated with uterine residual lesions, but 60% of the patients with residual lesions were menopausal, and 64% were older than 50 years. This could be attributed to the following reasons. When the patient is in menopause, the transformation zone area shrinks towards the cervical canal, resulting in the lesion being hidden, which is not conducive to direct observation and ultimately cannot be adequately excised [5, 16, 22]. Second, our patients underwent the LEEP rather than cold-knife conization (CKC). Previous studies have verified that the volume and depth of LEEP excised specimens are significantly smaller than those of excised CKC specimens [23, 24]. At the same time, the most important reason was that we did not conduct iodine tests or acetic acid tests before performing the LEEP, so the scope of the lesion could not be well observed. In subsequent LEEP, it is recommended that all patients undergo iodine or acetic acid tests to determine the specific location and extent of the lesions [25]. These findings may explain the increased percentage of positive margins in this study.

In contrast to previous studies [26], our study revealed that regardless of the type of incisal margin, it can affect residual uterine lesions. However, different kinds of incisal positive margins did not affect the residual lesions. Among the patients with residual uterine lesions, the ecto-cervical margin was positive in 16% (12/75), the stromal margin was positive in 29.3% (22/75), and the endo-cervical margin in 41.3% (31/75). The high proportion of positive endo-cervical margins possibly due to the older mean age of our enrolled patients. We also found that 14 patients (6.6%) with residual uterine lesions had negative margins. In addition, the relative risk of residual high-grade CIN in women with positive margins was 5 times higher than that in women with negative margins, which is consistent with previous studies [10]. In our study, 31.8% (43/137) of patients with free residual uterine lesions had positive endo-cervical margins, 19.0% (26/137) had positive stromal margins, and 11.7% (16/137) had positive ecto-cervical margins. Therefore, patients with positive margins should be treated with caution in subsequent clinical treatment. Not all patients with positive incisal margins need total hysterectomy. However, Ciavattini’s study noted that the proportion of menopausal patients who underwent hysterectomy for CIN increased annually [27]. This annual increase could have been caused by the coronavirus disease; our people have adopted a more radical approach to the treatment of the disease; similar to patients with positive incisal margins after the LEEP, and many people choose total hysterectomy mainly for the following reasons. First, because of a lack of understanding of the disease, patients may fear the disease, resulting in anxiety. Moreover, during long-term outpatient follow-up, patients experience psychological stress induced by diagnosis and treatment of the disease. Therefore, patients desired an immediate result [27]. The second group included patients with other diseases requiring surgical treatment, such as uterine fibroids and adenomyopathy [25]. Finally, a small number of menopausal patients had cervical atrophy, and there was no chance to perform the LEEP; therefore, patients demanded total hysterectomy. As mentioned above, 60% of the patients with residual lesions were menopausal women, including 5 patients with carcinoma in situ, which was the only 5 cases of cancer included in this study. Therefore, hysterectomy may be a more appropriate treatment for menopausal patients with risk factors. Young people, who wish to preserve fertility function, they can choose to follow up or undergo repeated excision.

Previous studies reported that glandular involvement (GI) was closely related to postoperative recurrence of the LEEP, and the number of patients with high-grade lesions showing glandular involvement was 4 times higher than that of patients with low-grade lesions [28–32]. However, on the contrary, some studies have confirmed that GI status did not affect the residual lesions in patients after excision [33, 34]. GI in our patients was found via colposcopic biopsy pathology, not via LEEP specimens, so for patients with glandular involvement, the scope of LEEP excision was increased. Moreover, Kim’s study confirmed that patients with GI who were diagnosed by colposcopic biopsy had a deeper LEEP depth (11 mm vs. 8 mm, *p* = 0.024) and a significantly reduced margin positivity rate [34]. This finding is consistent with our findings that GI is a protective factor for residual uterine lesions. Therefore, colposcopic pathology is necessary to indicate the presence or absence of glandular involvement.

One study verified that endocervical curettage (ECC) was more likely to reveal ≥ CIN2 lesions in women with ASC-US or LSIL cervical cytology or in an unsatisfactory colposcope examination [35]. According to current guidelines, when colposcopy is inadequate, ECC can be considered for nonpregnant patients [36]. Feng’s study showed that the detection rate of HSIL increased by 5% in patients who underwent colposcopy combined with ECC [37], and this detection rate increased with age [38]. At the same time, Feng’s study confirmed that 2 years after the LEEP, the rate of recurrence in patients with ECC positivity was as high as 15.9% [37]. Our study revealed that ECC suggesting HSIL was an independent risk factor for uterine residual lesions. The risk of uterine residue in ECC-positive patients is 2 times higher than that in ECC-negative patients. Based on the above studies, ECC-positive patients should carefully select postoperative treatment after a LEEP. However, our study did not involve a correlation analysis of LEEP combined with ECC, which is a major limitation of our study because ECC after LEEP is more important for the prediction of residual uterine lesions.

Studies have indicated that CIN is an independent risk factor for VaIN [39], and the incidence of VaIN in patients undergoing total hysterectomy due to HSIL was 7.4% [13]. Our study revealed that VaIN was present in only 15 of 212 patients and was detected during colposcopy, including 7 in VaIN1, 7 in VaIN2-3 and 1 carcinoma in situ. In addition, our study found that all vaginal lesions were located in the upper one-third of the vagina. However, it is interesting to note that VaIN was a protective factor for residual uterine lesions in this study, possibly because most of our patients underwent physical therapy for VaIN during the LEEP period. In addition, the scope of total hysterectomy was not sufficient (the vaginal wall lesions in this study were located in the upper one-third of the vagina, and most of them were located in the fornix, which was not removed during hysterectomy), resulting in the failure to find residual lesions; therefore, it was a protective factor against residual uterine tissue. We should also pay attention to the difficulty of vaginal exposure and various vaginal folds, which can result in missed diagnoses of vaginal lesions. We cannot rule out the possibility of such missed diagnosis in this study. Several studies have also suggested multipoint vaginal wall biopsy for patients with abnormal LCT but normal biopsy [40]. This provides us with an idea that physical therapy combined with a LEEP or improving the scope of hysterectomy may be a new treatment model. However, relevant studies are still needed to verify this.

Our multivariate analysis ruled out the effect of HPV infection on residual lesions. However, we found that older patients were more likely to develop non-16/18 HPV infections (*p* = 0.013, OR = 1.046, 95% CI: 1.010–1.084). Giannella’s study revealed that the non-16/18 and non-9-valent-vaccine types were rare in CIN3 patients younger than 30 years old. Moreover, there was a positive trend with increasing age in non-HPV-16/18 CIN3 patients [41]. Hence, in older women, non-16/18 HPV infections should be considered more seriously.

In this study, positive incisal margins and ECC (≥ CIN2) were risk factors for residual lesions, while glandular involvement and VaIN were protective factors. This finding suggested that we removed a larger area of tissue when the LEEP was performed in patients with glandular involvement, thus reducing the occurrence of residual uterine lesions. This means that colposcopic pathology needs to emphasize glandular involvement. When colposcopy also reveales vaginal intraepithelial lesions, the LEEP combined with physical vaginal wall therapy or expansion of the scope of the hysterectomy (including the partial vaginal wall) could be used to reduce residual uterine lesions. Although our study pointed out that there was little relationship between menopause and residual lesions, 60% of the patients with residual uterine lesions were menopausal patients, and all patients with cervical carcinoma in situ in this study were menopausal patients. Therefore, total hysterectomy may be a better choice for treating CIN in menopausal patients. However, for patients < 25 years or those who are concerned about the effect of treatment on future pregnancy outcomes, observation or repeat excision are recommended [42].

## Strengths and limitations

Most of the patients in this study were perimenopausal patients and were prone to CIN2-3. In addition, most patients underwent surgery within 6 months. During this time, there is almost no possibility of disease recurrence or progression, which further defines the residual lesion (it is the initial lesion of the cervix). In addition, we included age, menopausal status, ECC, incisal margin, vaginal intraepithelial lesions, and glandular involvement factors in one study for both univariate and multivariate analyses. This study emphasized that the colposcopic pathology needs to include glandular involvement. This study also provides some suggestions for the management of CIN2-3 in menopausal patients.

Nonetheless, our study was retrospective, the sample size was not large enough, and cervical glandular epithelial lesions were not included. Cervical glandular epithelial lesions are more likely to recur and metastasize, which is related to HPV18 [43]. Vaginal intraepithelial lesions have not been studied systematically.

## Data Availability

This study was performed in accordance with the Declaration of Helsinki and was approved by the Ethics Committee of the International Peace Maternity and Child Health Hospital in Shanghai. The requirement for informed consent was waived by the Ethics Committee of the International Peace Maternity and Child Health Hospital in Shanghai because of the retrospective nature of the study.
